# Charge Carriers Relaxation Behavior of Cellulose Polymer Insulation Used in Oil Immersed Bushing

**DOI:** 10.3390/polym14020336

**Published:** 2022-01-15

**Authors:** Yu Shang, Qiang Liu, Chen Mao, Sen Wang, Fan Wang, Zheng Jian, Shilin Shi, Jian Hao

**Affiliations:** 1 Shaanxi Electric Power Research Institute, State Grid Shaanxi Electric Power Co., Xi’an 710100, China; shangyu@dky.sn.sgcc.com.cn (Y.S.); liuqiang@dky.sn.sgcc.com.cn (Q.L.); maochen@dky.sn.sgcc.com.cn (C.M.); wangsen@dky.sn.sgcc.com.cn (S.W.); wangfan@dky.sn.sgcc.com.cn (F.W.); 2 State Key Laboratory of Power Transmission Equipment & System Security and New Technology, Chongqing University, Chongqing 400044, China; 202011021126t@cqu.edu.cn (S.S.); haojian2016@cqu.edu.cn (J.H.)

**Keywords:** cellulose polymer insulation, polarization/depolarization current, charge carrier, moisture, molecular dynamics simulation

## Abstract

Cellulose polymer insulation material is widely used in oil immersed bushing. Moisture is one of the important reasons for the deterioration of cellulose polymer insulation, which seriously threatens the safe and stable operation of bushing. It is significant to study the polarization and depolarization behavior of oil-immersed cellulose polymer insulation with different moisture condition under higher voltage. Based on polarization/depolarization current method and charge difference method, the polarization/depolarization current, interfacial polarization current and electrical conductivity of cellulose polymer under different DC voltages and humidity were obtained. Based on molecular-dynamics simulation, the effect of moisture on cellulose polymer insulation was analyzed. The results show that the polarization and depolarization currents become larger with the increase in DC voltage and moisture. The higher applied voltage will accelerate the charge carrier motion. The ionization of water molecules will produce more charge carriers. Thus, high DC voltage and moisture content will increase the interface polarization current. Increased moisture content results in more charge carriers ionized by water molecules. In addition, the invasion of moisture will reduce the band width of cellulose polymer and enhance its electrostatic potential, so as to improve its overall electrical conductivity. This paper provides a reference for analyzing the polarization characteristics of charge carriers in cellulose polymer insulation.

## 1. Introduction

The oil-immersed transformer is the core equipment of power transmission in the power grid, wherein safety and stability are significantly important to energy security and social stability. According to statistics, fault of bushing is one of the main causes for transformer faults, and the cellulose polymer paper, as the main insulation material of bushing, is the main factor contributing to the fault of bushing after moisture invaded [1,2,3]. To ensure the safe operation and stability of the transformer, it is necessary to analyze the time-domain relaxation behavior of carriers at high applied voltage with various moisture contents.

To date, some scholars have studied the effect of moisture on cellulose polymer paper. Wenyu Ye, et al. found that the structure of liquid-solid interface is determined by the interaction between insulating oil and cellulose polymer paper, which is based on the Van der Waals effect, and water molecules will gather at the interface because of the interaction of the liquid-solid interface at certain electric field value [4]. Guanwei Long, et al. have simulated the distribution of H+ and OH− for oil and cellulose polymer paper insulation when moisture invaded, explaining the effect of moisture on the cellulose polymer paper and oil insulation from a microscopic point of view [5]. Haoxiang Zhao et al. found that when charge carriers pass through the interface between cellulose polymer paper and oil, the insulating paper has an obstruction effect, while the existence of water molecules would produce more charge carriers and reduce the obstruction effect, resulting in lower conductivity of cellulose polymer paper and different dielectric response characteristics [6]. The above research shows that moisture will have a great influence on the dielectric properties of cellulose polymer paper.

In order not to damage the sealing performance of bushing, nondestructive testing methods based on dielectric relaxation theory have been widely used by scholars, among which polarization and depolarization current (PDC) method and frequency domain spectroscopy (FDS) method are the most popularly used [7,8,9]. Both methods have the advantages of simple operation and rich insulation information. However, FDS is usually used to study the dielectric response characteristics at different frequencies, focusing on the analysis of dielectric constant and dielectric loss factor. While PDC is used to analyze the charge accumulation and charge carrier relaxation behavior of cellulose polymer paper because it can measure the time domain dielectric response characteristics of insulating oil and cellulose polymer paper. Quanmin Dai et al. found that the dielectric response of bushing changes when moisture invaded, which provided a basis for judging the moisture content of cellulose polymer paper by PDC [10]. Feng Yang tested the cellulose polymer paper by PDC and found that the polarization and depolarization current increases with higher moisture content; they established a relationship between the moisture content of bushing and the PDC results [11]. T. K. Saha et al. proposed a method to calculate the conductivity of cellulose polymer paper by the polarization and depolarization current, providing a method to analyze charge accumulation by conductivity [12].

Although some scholars have studied the correlation between the dielectric characteristic parameters of bushing insulating paper in time domain and moisture, most of the research is based on low excitation voltage, resulting in low signal-to-noise ratio, which is easy to be interfered by environmental noise. Additionally, and yet worse, the cellulose paper in the bushing works at a higher voltage, which means the traditional low-voltage measurement results and rules are not applicable to a higher excitation voltage. In addition, there is little research on analyzing the charge accumulation at the interface of oil-paper insulation in bushing based on PDC measurement results. Therefore, it is necessary to analyze the time-domain relaxation behavior of the bushing insulating paper with various moisture contents at high voltage.

This paper studies the time domain relaxation behavior of charge carriers for cellu-lose polymer insulation used in oil immersed bushing under higher voltage. Firstly, the polarization and depolarization currents of cellulose polymer insulation with different moisture conditions are obtained. Then, the influence of moisture and applied voltage on the characteristics of interface polarization behavior is studied; the motion of the interface charge carriers during the process of polarization and depolarization and the relationship between the steady state time of electrical conductivity are analyzed. Finally, based on molecular dynamics simulation, the effect of moisture on the insulation properties of cel-lulose polymers is analyzed.

## 2. Experiment and Theoretical Analysis

### 2.1. Experiment

**Figure 1:**
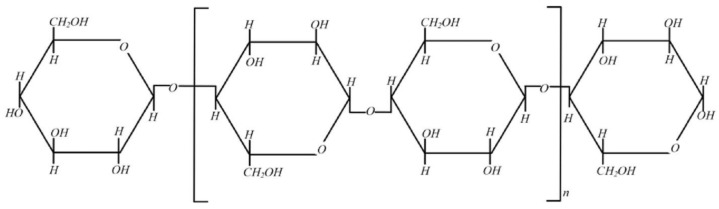
Chemical structure of cellulose macromolecular chain.

Cellulose is a natural polymer compound, its chemical structure is a linear polymer composed of many β-D-glucopyranosyl groups connected to each other by 1, 4-β glycoside bonds. Cellulose polymer insulation is mainly composed of cellulose macromolecular chains composed of cellulose monomers, as shown in Figure 1. Because cellulose polymer insulation has excellent insulation performance, it is widely used in the field of high voltage insulation. The chemical structure of cellulose macromolecular chain is shown as Figure 1 [13,14].

**Figure 2:**
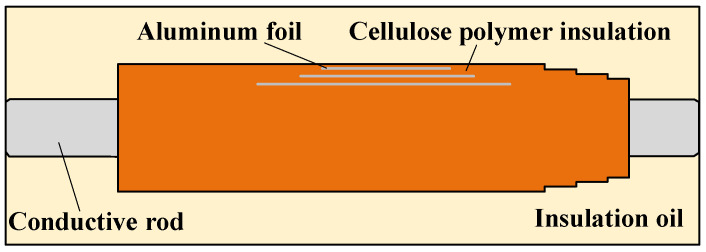
Structure diagram of oil immersed bushing.

In this paper, an oil immersed bushing with cellulose polymer insulation, where the maximum voltage is 40.5 kV, is tested. The structure diagram of the bushing is shown in Figure 2. The insulation performance of this bushing may be reduced due to moisture in the oil and cellulose polymer insulation. In order to study the influence of moisture on the time domain relaxation behavior, the cellulose polymer insulation and insulation oil are treated with moisture absorption before the bushing is manufactured and packaged.

**Figure 3:**
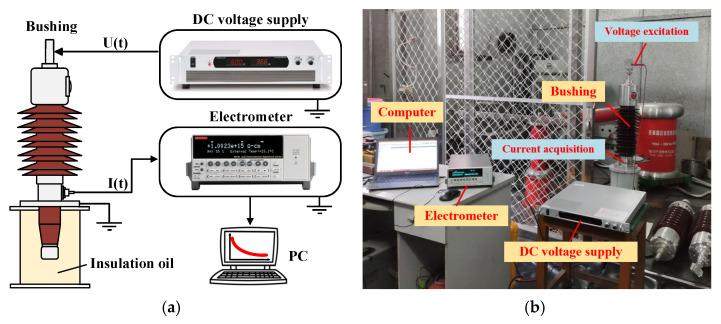
PDC test. (**a**) Schematic diagram; (**b**) Physical diagram.

PDC tests in different DC voltages are carried out; the schematic diagram and physical diagram of PDC test is shown in Figure 3. In this paper, the DC high voltage power supply(AU-20*60, Matsusada, Osaka, Japan) is used to excite the bushing, and the high-precision electrometer(6517 B, Keithley, OH, USA) is used to collect the current signal at the end screen of the bushing. The polarization/depolarization process of the bushing cellulose polymer can be further explained by analyzing the changes in the excitation voltage and response current. The charge and discharge process for bushing lasts for 3000 s, and the applied voltages are 200 V, 500 V, 1000 V, 2000 V and 4000 V, respectively, and the environmental temperature and relative humidity are 20 °C and 62%, respectively.

### 2.2. Polarization/Depolarization Principle and Charge Analysis for Composite Polymer Medium

**Figure 4:**
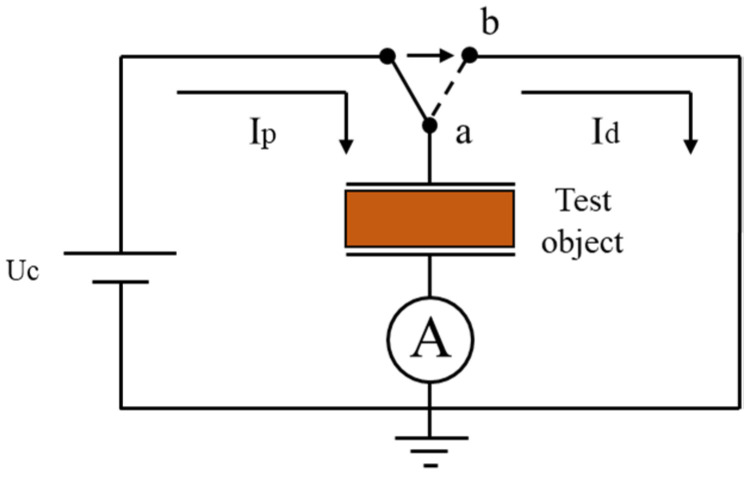
Schematic diagram.

By applying and removing DC voltages to the insulating medium, the dielectric response characteristics in time domain from the polarization and depolarization current can be extracted based on a PDC test. The schematic diagram is shown as Figure 4. By connecting the switch to point a and then point b, the insulating medium is charged and then discharged, these two processes are polarization and depolarization processes of the insulating medium, respectively, and the current generated in these two processes are called polarization current $i_{pol}$ and depolarization current $i_{depol}$ , correspondingly.

For the insulating medium, the polarization process mainly includes dipole polarization and interface polarization. Due to the difference of dielectric constant and electrical conductivity of composite insulating mediums, the free charge will accumulate at the interface and will not dissipate easily, resulting in the asymmetry of polarization process and depolarization process. Polarization current consists of dipole relaxation current $i_{conductance}$ , dipole polarization current $i_{dipole-pol}$ and interface polarization current $i_{interface-pol}$ . The depolarization current mainly consists of dipole relaxation current $i_{dipole-depol}$ . In general, the dipole polarization current is equal to dipole relaxation current. The polarization current and depolarization current are shown in Equations (1) and (2).

(1) $$i_{pol}=i_{dipole-pol}+i_{conductance}+i_{interface-pol}$$

(2) $$i_{depol}=i_{dipole-pol}=i_{dipole-depol}$$

Electrical conductivity can reflect the degree of moisture content of insulating materials. Charge difference analysis method (CDA) can effectively calculate the electrical conductivity of insulating materials by analyzing the change characteristics of the charge amount difference in charge and discharged process [15,16,17,18]. By integrating the difference of polarization and depolarization current curve over time, charge amount difference can be obtained, as shown in Equations (3) and (4) [19,20].

(3) $$q_{pol}\left(t_{i}\right)=\sum \left[i_{pol}\left(t_{i}\right)\times t_{i}\right]$$

(4) $$q_{depol}\left(t_{i}\right)=\sum \left[i_{depol}\left(t_{i}\right)\times t_{i}\right]$$

Thus the charge amount difference at any time is as Equation (5), and the difference between polarization current and depolarization current at any time is as Equation (6):

(5) $$\Delta Q\left(t_{i}\right)=\sum i_{dc}\left(t_{i}\right)\times t_{i}$$

(6) $$i_{dc}\left(t_{i}\right)=i_{pol}\left(t_{i}\right)-i_{depol}\left(t_{i}\right)$$

The charge amount difference is numerically the integral of the conductance current over time. As the conductance current changes little with time, the slope *k* of charge amount difference function with time is approximately constant, as shown in Equation (7).

(7) $$k=\frac{\left[q_{pol}\left(t_{2}\right)-q_{depol}\left(t_{2}\right)]-[q_{pol}\left(t_{1}\right)-q_{depol}\left(t_{1}\right)\right]}{t_{2}-t_{1}}$$

From Equations (6) and (7), $i_{dc}\left(t_{i}\right)$ can be expressed as Equation (8):

(8) $$i_{dc}\left(t_{i}\right)=\frac{q\left(t_{i}\right)}{t_{i}}=k$$

When the time of polarization and depolarization process is equal and long enough, the current difference at the final moment is equal to the conductance current, as shown in Equation (9). The electrical conductivity *σ_r_* of the composite insulating medium can be obtained from the conductance current, as shown in Equation (10).

(9) $$i_{dc}\left(t_{final}\right)=i_{pol}\left(t_{final}\right)-i_{depol}\left(t_{final}\right)=i_{conductance}$$

(10) $$\sigma_{r}\approx \frac{\varepsilon_{0}}{C_{0}U_{0}}\left[i_{pol}\left(t_{final}\right)-i_{depol}\left(t_{final}\right)\right]=\frac{\varepsilon_{0}i_{conductance}}{C_{0}U_{0}}$$

### 2.3. Electrical Conductivity Calculation for Cellulose Polymer Insulation

**Figure 5:**
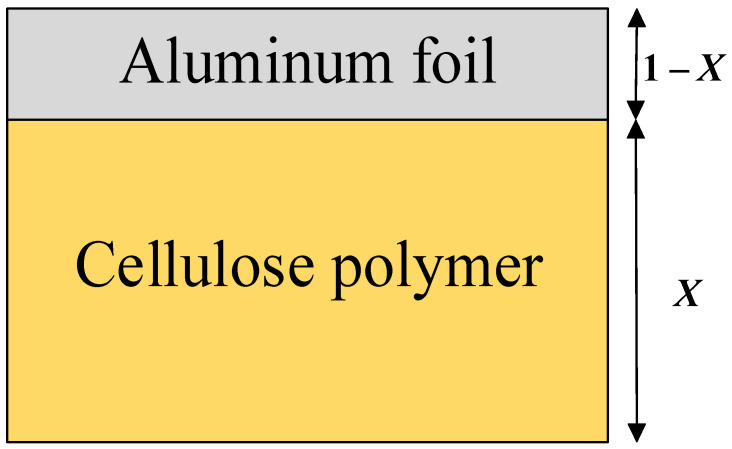
*X* model of oil immersed cellulose polymer insulation.

The composite insulation structure in the bushing is composed of aluminum foil and oil immersed cellulose polymer wrapped closely, as shown in Figure 5. The electrical conductivity of the cellulose polymer insulation can be obtained by using simplified *X-Y* model when the electrical conductivity of the whole insulating material is known [20].

As for simplified *X* model, the electrical conductivity of the composite insulating structure is shown as Equation (11), where *X* is the thickness ratio.

(11) $$\sigma_{r}=\frac{\sigma_{cellulose}\sigma_{Al}}{\sigma_{cellulose}\left(1-X\right)+\sigma_{Al}X}$$

Since the electrical conductivity of aluminum foil is much greater than that of cellulose polymer and the thickness of aluminum foil is negligible compared with that of cellulose polymer insulation, which means *X* equals to 1, the electrical conductivity of cellulose polymer insulation can be simplified, and the electrical conductivity of the cellulose polymer is as shown in Equation (12).

(12) $$\sigma_{cellulose}=\frac{\varepsilon_{0}k}{C_{0}U_{0}}$$

### 2.4. Modeling of Molecular Dynamics Simulation

In order to study the effect of moisture on the insulation properties of cellulose polymer, the models of cellulose without moisture and after moisture were constructed by using Materials Studio software [21].

**Figure 6:**
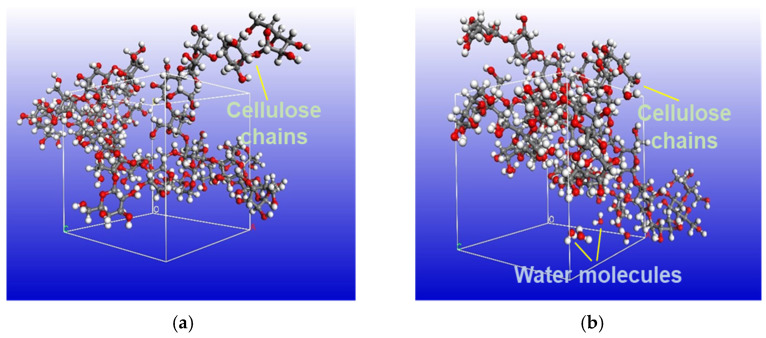
The two cellulose polymer insulation models: (**a**) Cellulose model; (**b**) Cellulose model with 3.5% water.

Three cellulose chains with a degree of polymerization of 10 were used as the cellulose polymer insulation model without moisture, while 3.5% water molecules were added into three cellulose chains with a degree of polymerization of 10 as the cellulose polymer insulation model with moisture. The two cellulose polymer insulation models are shown in Figure 6.

First, 10,000 steps of geometric optimization were carried out for the two models by using the Steepest descent method. Then, the two models were annealed to make the model reach the most realistic condition; the annealing temperature was 300–500 K. Under Compass force field, constant-pressure and constant-temperature ensemble (NPT ensemble), with a constant number of molecules, pressure, and temperature, was used to balance each model and to make the model more reasonable with 500 ps. Based on the density functional theory (DFT), the calculations for two cellulose models were analyzed by DMol3 Tool in Materials Studio. The geometry optimization, molecule orbitals and electrostatic potential were calculated by employing the PBE function under the generalized gradient approximation (GGA) exchange-correlation term. The double numerical plus polarization (DNP) basis set was applied for setting the parameters of C, H, O atoms in this computational work. The all-electron method was adopted for core electron calculation.

## 3. Results

### 3.1. Polarization and Depolarization Current Characteristics for Cellulose Polymer Insulation

**Figure 7:**
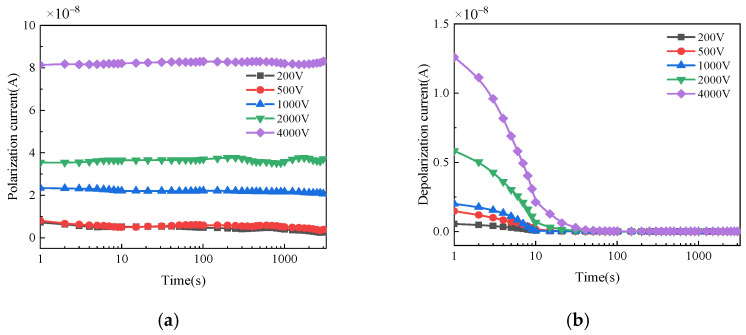
Polarization/depolarization current of normal bushing: (**a**) Polarization current; (**b**) Depolarization current.

**Figure 8:**
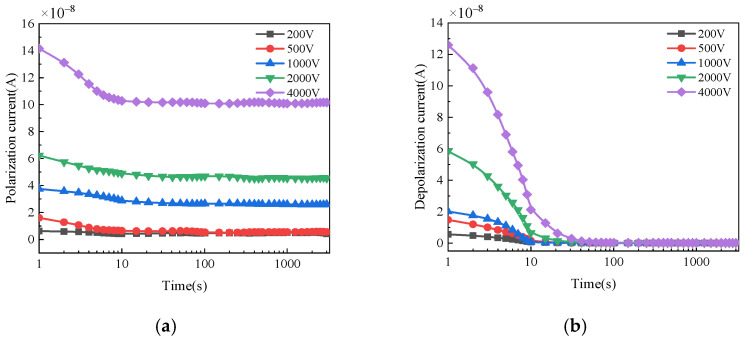
Polarization/depolarization current of bushing with damped oil: (**a**) Polarization current; (**b**) Depolarization current.

**Figure 9:**
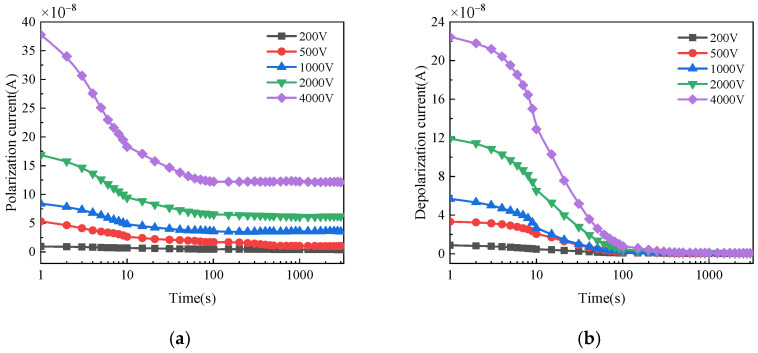
Polarization/depolarization current of bushing with damped cellulose polymer insulation: (**a**) Polarization current; (**b**) Depolarization current.

The polarization and depolarization current of bushings with different moisture conditions and applied voltages are shown in Figure 7, Figure 8 and Figure 9. For normal bushing, the polarization current will hardly change significantly with time under any polarization voltage. The depolarization current decreases rapidly to a stable value in the first 10 s. For dampened oil bushing, under any polarization voltage, the polarization current decreases slowly in the first 10 s, and then tends to be almost stable. The depolarization current decreases rapidly in the first 50 s, and with the passage of time, the decreasing trend gradually slows down and finally tends to be stable. For dampened cellulose polymer bushing, under any polarization voltage, the polarization current will gradually decrease with time in a short time, and then gradually tend to be stable. The depolarization current decreases rapidly in the first 100 s, and with the passage of time, the decreasing trend gradually slows down and finally tends to be stable.

This phenomenon can be explained by the differing hydrophilic qualities of oil and cellulose polymer. Since the hydrophilic quality of cellulose polymer is much higher than that of insulation oil, when water is present, most water molecules in the insulation oil will migrate to the cellulose polymer with the invasion of moisture. As polar molecules, the more moisture infiltrates into the cellulose polymer insulation, the more polar molecules are involved in the polarization reaction, leading to a larger polarization intensity difference. The moisture content, on one hand, can increase the electrical conductivity of the insulation system, whereas, on the other hand, it can enhance the response speed of the interface polarization, aggravating the dielectric asymmetry of the oil-immersed cellulose polymer insulation system at the same time. Finally, the insulation resistance and capacitance increase, and the polarization process is aggravated [22,23,24]. Thus, the polarization and depolarization current become larger when the moisture content increases at the same applied voltage.

When the applied voltage is lower than 1000 V, the current fluctuates significantly with time. This is because the polarization process of oil-immersed cellulose polymer insulation system inside the bushing cannot be fully stimulated by low DC voltage in such a large size. Therefore, the polarization and depolarization currents of the insulation are small and susceptible to the interference of environmental noise, which has an obvious influence on the accuracy of the test results. When the applied DC voltage increases, the corresponding current becomes larger and the fluctuation of that decreases. The development trend of polarization and depolarization current is not affected by applied DC voltage, which means the applied DC voltage has little influence on the general trend of the dielectric polarization process. Thus, a high applied DC voltage test can not only improve the signal-to-noise ratio, but also reflect the polarization process of bushing more clearly, resulting in a more accurate and effective test result.

### 3.2. Interface Polarization Characteristics of Cellulose Polymer Insulation

**Figure 10:**
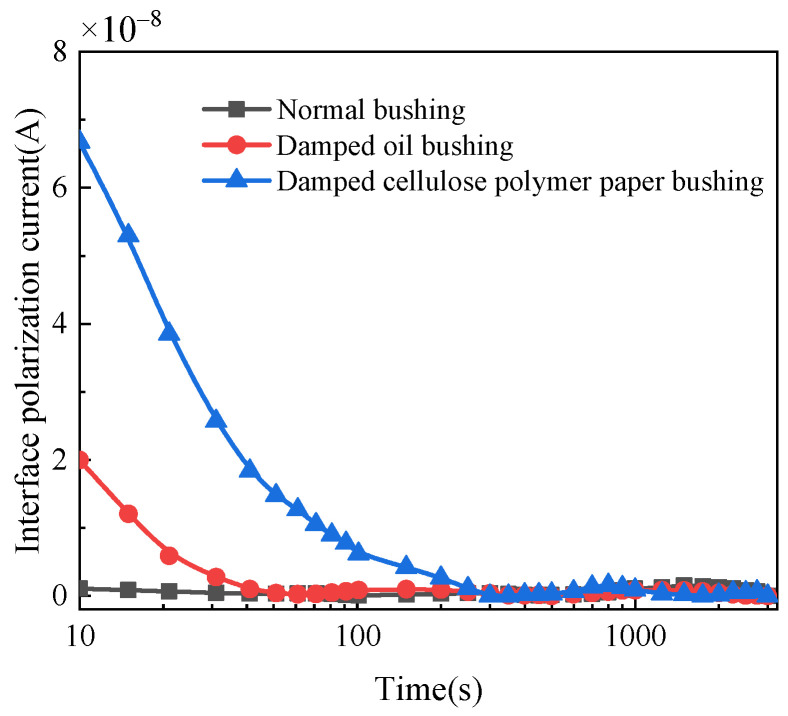
Interface polarization current of bushings with different moisture conditions.

**Figure 11:**
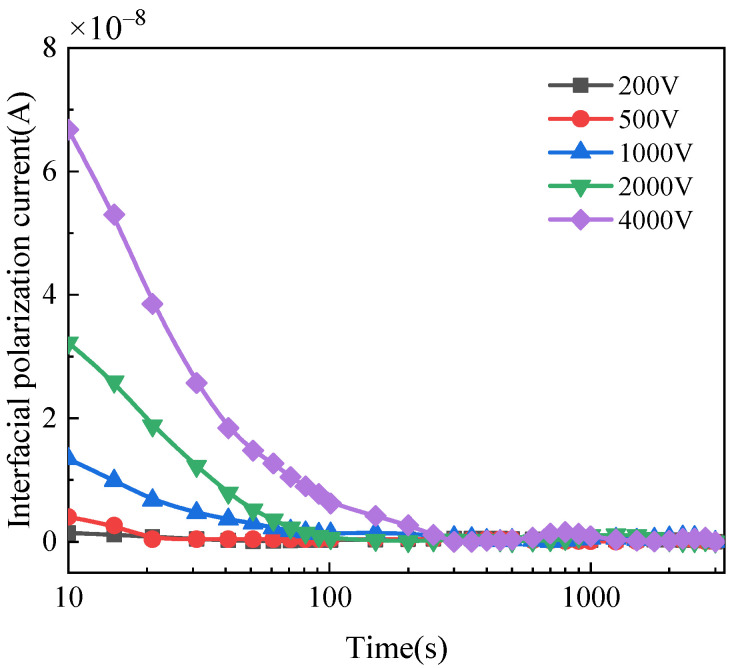
Interface polarization current of dampened cellulose polymer insulation in bushing under different DC voltages.

The interface polarization current of the three bushings can be calculated by Equations (1)–(3), as shown in Figure 10 and Figure 11. Figure 10 shows that the interface polarization current of the bushing with different moisture condition decreases to zero with time. While for normal bushing, the interface polarization current does not change significantly and always tends to be zero. Compared with dampened oil bushing, the initial interface polarization current of the bushing with dampened cellulose polymer insulation is larger and takes longer time to decay to zero. Figure 11 reflects that with the increase in applied DC voltage, the interface polarization current increases, and the current decreases slowly with the time.

**Figure 12:**
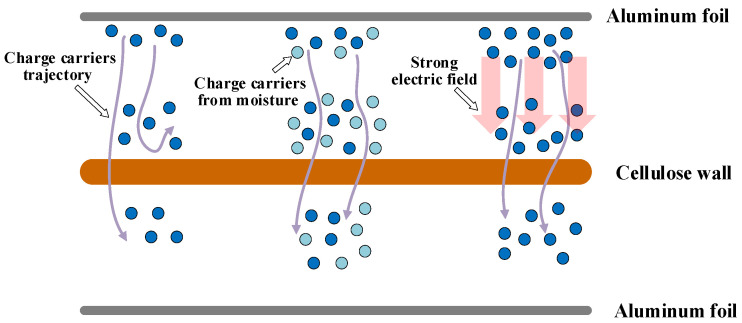
Migration process of charge carriers at the cellulose polymer interface.

The insulation system of the bushing is composed of aluminum foil and cellulose polymer, and the dielectric constants of both are different. When applying DC voltage in the insulation system, the interface polarization process occurs, and an interface polarization current is generated. When the voltage increases, the electric field becomes larger, accelerating the motion of the charge carriers. When the moisture infiltrates into the insulation medium, the water molecules are ionized to produce more charge carriers under the effect of electric field. This explains the characteristic of interface polarization current under the influence of moisture content and applied DC voltage. The migration process of charge carriers at the cellulose polymer interface is shown in in Figure 12.

### 3.3. Charge Carriers and Electrical Conductivity Analysis of Cellulose Polymer Insulation

**Figure 13:**
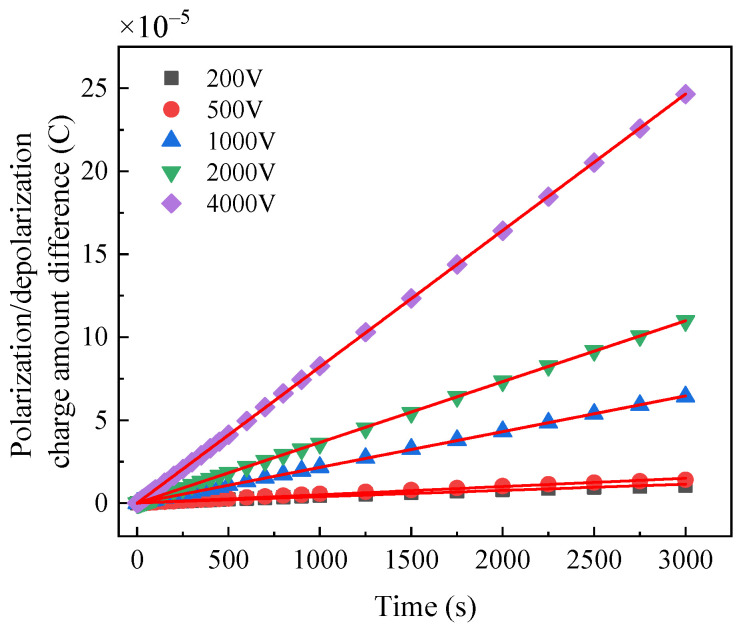
Polarization/depolarization charge amount difference curve of normal bushing.

**Figure 14:**
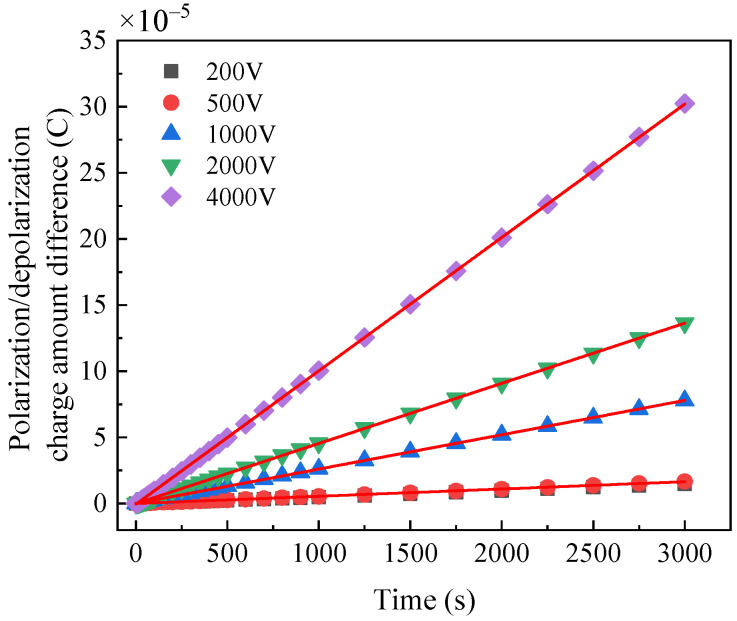
Polarization/depolarization charge amount difference curve of dampened oil bushing.

**Figure 15:**
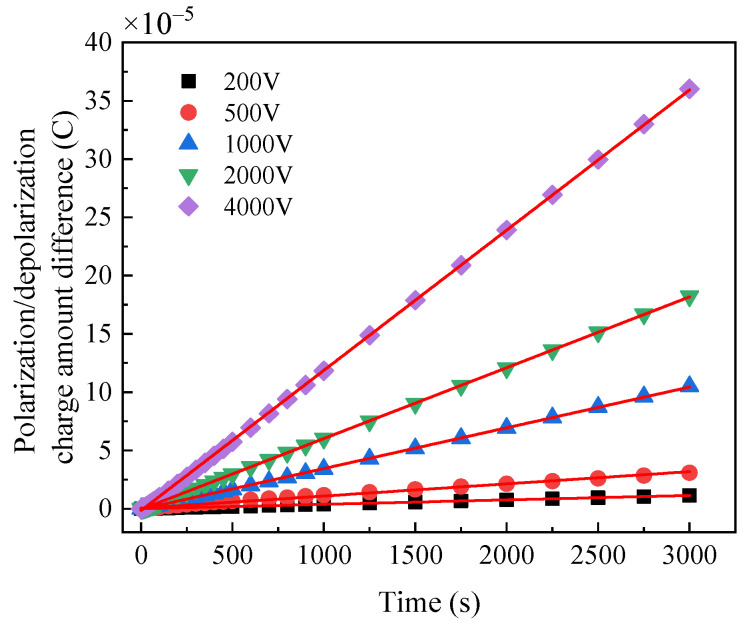
Polarization/depolarization charge amount difference curve of damped cellulose polymer paper bushing.

The charge difference spectrum of normal bushing, dampened oil bushing and dampened cellulose polymer paper bushing under different voltages are shown in Figure 13, Figure 14 and Figure 15, respectively. The results show that the charge difference curves of the three bushings have similar characteristics. According to Equation (8), the slope of the charge difference curve is approximately equal to the conductance current, indicating that the accumulation rate of the charge difference tends to be constant. With the increase in the excitation voltage, the faster is the accumulation rate of the charge difference. However, under the same excitation voltage, the slope of the charge difference curve of the normal bushing is always less than that of the moistened bushing, indicating that water will also affect the accumulation rate of the charge difference.

**Figure 16:**
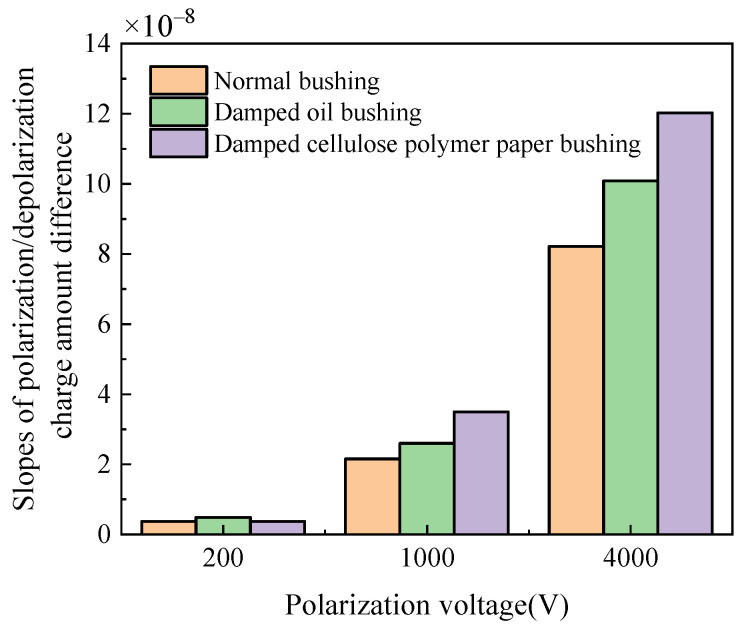
Slopes of charge amount difference curve at different applied DC voltages.

The slopes of the polarization/depolarization charge amount difference at 200 V, 1000 V and 4000 V are shown in Figure 16. It is indicated that the slopes at larger applied DC voltage are much higher and more distinguished than those at lower applied DC voltages, which indicates that the moisture condition can be confirmed by the slope of charge amount difference curve at high applied DC voltage in PDC test.

**Figure 17:**
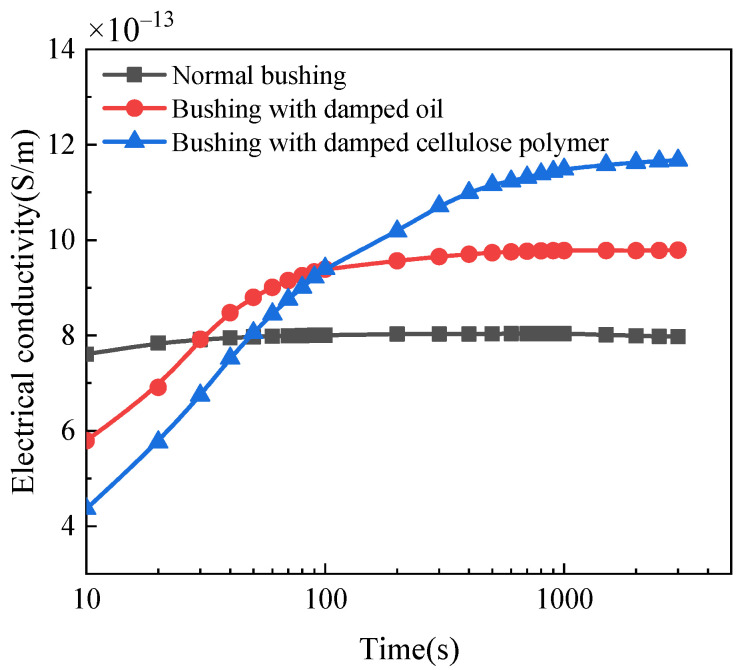
Electrical conductivity change behavior of normal bushing, bushing with dampened oil and dampened cellulose polymer insulation.

The electrical conductivity change behavior of cellulose polymer insulation with different moisture conditions is shown in Figure 17. It is shown that the electrical conductivity increases with time and tends to be stable after 1000 s. The stable value of electrical conductivity is the real electrical conductivity *σ*. The geometric capacitance of the insulation in the bushing is 228 pF, thus the real electrical conductivity of normal, bushings with dampened oil and dampened cellulose polymer insulation are 7.9748 × 10^−13^ S/m, 9.7884 × 10^−13^ S/m and 1.1672 × 10^−12^ S/m, respectively. The results indicate that electrical conductivity increases when the moisture content in the insulation material is higher, leading to the deterioration of the insulation performance.

For normal bushing, electrical conductivity is stabilized the fastest because the interface polarization current dissipates faster with time, resulting in the electrical conductivity current taking less time to stabilize. However, the polarization process can be influenced by the invasion of moisture when the insulation material is dampened, resulting in a longer time that the interface polarization current dissipates and the higher value of the interface polarization current. Additionally, moisture content can also lead to a longer time that the conductance current takes to be stable and can decrease the initial value of polarization and depolarization charge amount difference. The polarization current increases significantly while the depolarization current is less affected under the influence of moisture content, leading to the increase in slope of polarization/depolarization charge amount difference. Because the electrical conductivity is linear with the slope of the polarization/depolarization charge amount difference curve, the electrical conductivity of the bushing with dampened oil and dampened cellulose polymer in the stable state is larger than that of normal bushing. These reasons explain that normal bushing has a larger electrical conductivity than that of bushing with dampened oil and cellulose polymer insulation, and that the electrical conductivity of normal bushing takes less time to stabilize.

### 3.4. Molecular Dynamics Analysis Based on Band Structure and Electrostatic Potential

**Figure 18:**
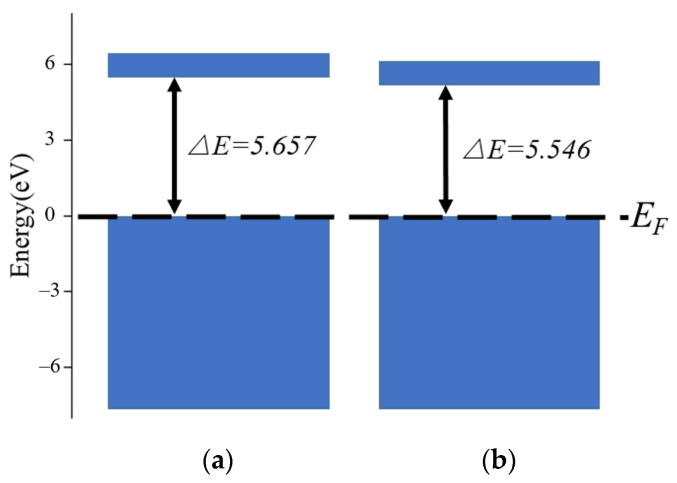
The band structure of two cellulose polymer insulation models: (**a**) Cellulose model; (**b**) Cellulose model with 3.5% water.

The band structure of unmoistened cellulose and moistened cellulose is shown in Figure 18. It can be seen that the band width (Δ*E*) of unmoistened cellulose is 5.657 eV, while the band gap width of moistened cellulose is 5.546 eV. The wider the band width, the weaker the electrical conductivity. The energy band width of cellulose is narrowed after the water content of cellulose is increased, so the moistened cellulose polymer insulation electrical conductivity is stronger.

**Figure 19:**
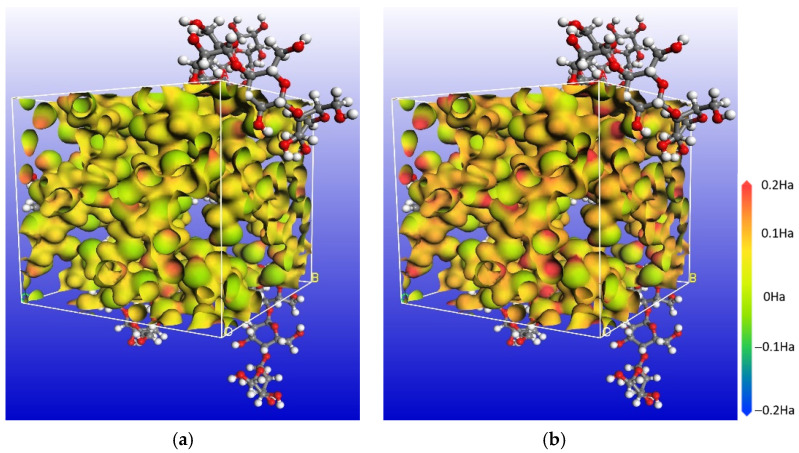
Electrostatic potential of two cellulose models: (**a**) Cellulose model; (**b**) Cellulose model with 3.5% water.

The electrostatic potential of unmoistened cellulose and moistened cellulose is shown in Figure 19. The red is the positive electrostatic potential, and the blue is the negative electrostatic potential. It can be seen that with the increase of water content in cellulose, water molecules would increase the positive electrostatic potential in the model, so the polarity of the model increases.

Due to the existence of a large number of hydroxyl groups in cellulose, these hydroxyl groups can easily form hydrogen bonds with each other. It is precisely because of the existence of hydrogen bonds that cellulose has strong intermolecular and intramolecular interaction forces, and the ability of cellulose to resist external damage is also closely related to the concentration of hydrogen bonds. The molecular simulation results show that the presence of water destroys the intramolecular and intermolecular hydrogen bonds in the cellulose chain, and forms a new hydrogen bond interaction with the oxygen atom on the cellulose hydroxyl group. Water mainly acts on the hydroxyl and glycoside bonds of cellulose and destroys their stability. Therefore, the motion of charge carriers in the insulating paper cellulose is more intense under the action of the electric field. This is consistent with the conclusion in [25] that charge carriers have a significant accelerating effect on the hydrolysis of cellulose.

In conclusion, with the invasion of moisture, the electrical conductivity and polarity of cellulose polymer insulation will increase. Under the excitation of DC electric field, the polarization/depolarization process is more intense, which is specifically reflected in the experiment by the polarization/depolarization current being greater. In addition, the electrostatic potential of cellulose polymer is stronger after moisture invasion, indicating that its electrical conductivity characteristics are better under the action of electric field, that is, the electrical conductivity is greater, which verifies the calculated electrical conductivity of cellulose polymer insulation in different oil-immersed bushings above.

## 4. Conclusions

This paper studies the time domain relaxation behavior of charge carriers for cellulose polymer insulation used in oil-immersed bushing, and the characteristics of dielectric response and motion of charge carriers have been analyzed. The conclusions drawn are as follows:

1. The polarization/depolarization current of bushings increases when the applied voltage becomes higher. High applied voltage can decrease the influence of noise. Moisture can aggravate the polarization/depolarization process, leading to higher polarization/depolarization current.
2. When applied voltage is higher, the speed of charge carriers increases, leading to the increase of interface polarization current. Water molecules are ionized under the effect of electric field, producing more charge carriers, thus increasing the interface polarization current as well. With the increase of interface current, the relaxation behavior will be further intensified.
3. The charge amount difference of polarization and depolarization current is linearly aligned with the time, and the slope becomes larger with the increase in moisture, which is more obvious under high applied voltage. Water molecules produce more charge carriers after being ionized and provide more paths for charge movement.
4. With the invasion of moisture, the band width of cellulose polymer insulation becomes narrower, and its electrostatic potential increases, which improves the electrical conductivity of cellulose polymer insulation, and the polarization characteristics under DC electric field are more significant.
