# Correction: Shang et al. Charge Carriers Relaxation Behavior of Cellulose Polymer Insulation Used in Oil Immersed Bushing. *Polymers* 2022, *14*, 336

**DOI:** 10.3390/polym14061206

**Published:** 2022-03-17

**Authors:** Yu Shang, Qiang Liu, Chen Mao, Sen Wang, Fan Wang, Zheng Jian, Shilin Shi, Jian Hao

**Affiliations:** 1 Shaanxi Electric Power Research Institute, State Grid Shaanxi Electric Power Co., Xi’an 710100, China; shangyu@dky.sn.sgcc.com.cn (Y.S.); liuqiang@dky.sn.sgcc.com.cn (Q.L.); maochen@dky.sn.sgcc.com.cn (C.M.); wangsen@dky.sn.sgcc.com.cn (S.W.); wangfan@dky.sn.sgcc.com.cn (F.W.); 2 State Key Laboratory of Power Transmission Equipment & System Security and New Technology, Chongqing University, Chongqing 400044, China; 202011021126t@cqu.edu.cn (S.S.); haojian2016@cqu.edu.cn (J.H.)

The authors wish to make the following corrections to this paper: [1].

**Error in Figure**

In the original publication, there was a mistake in **Figure 3. (b) “Voltage excitation**” **and “Current acquisition” are reversed.** The corrected **Figure 3b** appears below.

**Missing Funding**

Author would like to modify the funding section to:

**Funding:** This work is supported by State Grid Shaanxi Electric Power Co., Shaanxi Electric Power Research Institute (SGSNKYOOSPJS2000308, Time/Frequency Domain Dielectric Response Combined Diagnosis and Analysis Technology and Application Research on High Voltage Bushing Defects and Health Margin—Research on Combined Diagnosis Method of Dielectric Response of Bushing).

The authors apologize for any inconvenience caused and state that the scientific conclusions are unaffected. The original publication has also been updated.

## Figures and Tables

**Figure 3:**
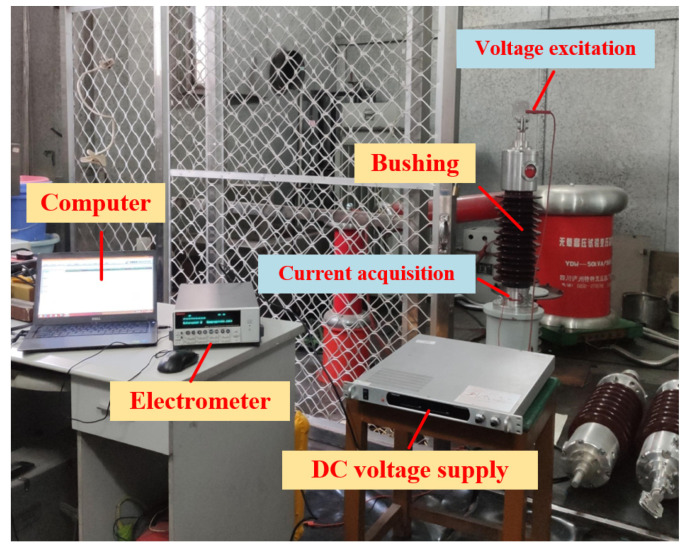
(**b**) Physical diagram.
